# Influence of a pediatric fruit and vegetable prescription program in Flint Michigan on caregiver perceptions of pediatric health care

**DOI:** 10.1016/j.pmedr.2026.103421

**Published:** 2026-02-20

**Authors:** Amy Saxe-Custack, Diana Haggerty, Gayle Shipp, Jenny LaChance

**Affiliations:** Charles Stewart Mott Department of Public Health, Michigan State University–Hurley Children's Hospital Pediatric Public Health Initiative, 200 E 1st Street, Flint, MI 48502, United States

**Keywords:** Fruits and vegetables, Produce prescriptions, Pediatrics, Nutrition, Primary care, Food is medicine

## Abstract

**Objectives:**

Pediatric clinics have introduced fruit and vegetable prescription programs to address health care disparities. This study assessed whether exposure to a fruit and vegetable prescription program that provided young patients with $15 produce prescriptions during office visits was associated with improvements in caregiver perceptions of pediatric care and if this differed by program engagement.

**Methods:**

This non-controlled longitudinal intervention trial included data from a consecutive sample of caregiver-child dyads at one pediatric clinic. Data were collected from February 2021 through May 2024. At baseline, 12-, and 24-months, caregivers completed the 23-item Parent's Perceptions of Primary Care measure which yielded a total score, and seven subscale scores dichotomized to evaluate probability of giving a maximum score. Program engagement was defined as redemption of at least one prescription. Marginal means and predicted probabilities of outcomes, linear mixed models and generalized linear mixed models were estimated.

**Results:**

247 dyads were included. Trends in overall satisfaction scores (p-interaction = 0.01) and subscales for communication (p-interaction = 0.06) and coordination of care (p-interaction = 0.02) increased among engaged participants and remained stable or decreased among non-engaged participants.

**Conclusions:**

This study suggests that pediatric fruit and vegetable prescription program engagement is positively associated with caregiver perceptions of pediatric health care.

## Highlights

• Parent-provider ties may be strengthened by produce prescription programs for youth.
• Produce prescription use is linked to improved perceptions of pediatric health care.
• Engagement in produce prescription programs is key to changing perceptions of care.

## 1 Introduction

Primary care physicians are the leading source of health information among young patients and their families and are well-positioned to address general health needs (Daniels and Hassink, 2015; Lupi et al., 2014; Shahidullah et al., 2023). In addition to providing sound medical advice, providers must also consider important factors that influence quality of primary health care, including access to appointments, social determinants of health screening and referral, and coordination of care. Unfortunately, research has documented disparities in pediatric health care across socioeconomic and racial groups (Chokshi, 2018; Flores et al., 2005; Seid et al., 2003; Shahidullah et al., 2023; Weech-Maldonado et al., 2001). These disparities in care may lead to preventable differences in outcomes with marginalized children facing disproportionate health burdens (Shahidullah et al., 2023). Recently, pediatric clinics across the United States (US) have introduced fruit and vegetable prescription programs (FVPPs) that are designed, in whole or in part, to address such disparities (Ridberg et al., 2019a).

Poor access to healthy foods too often prevents youth who live in low-income communities from meeting dietary guidelines (Baker et al., 2006; Morland et al., 2002; Walker et al., 2010; Zenk et al., 2011). FVPPs assist patients and families in accessing healthy foods and reducing food insecurity (Ridberg et al., 2019a; Saxe-Custack et al., 2021, Saxe-Custack et al., 2025). Additionally, produce prescriptions remind pediatricians to discuss the importance of nutrition during critical periods of growth and development, which may influence caregiver perceptions of their child's care. Similar to medical prescriptions, fruit and vegetable prescriptions are typically ordered by physicians and provided to patients to exchange for fresh produce at local farmers' markets or food retailers. Studies have documented the success of FVPPs in improving diet, food security, and clinical markers of health among pediatric patients (Muleta et al., 2024; Ridberg et al., 2019b; Saxe-Custack et al., 2019a, Saxe-Custack et al., 2021, Saxe-Custack et al., 2025). Moreover, pediatric FVPPs have shown promise in improving caregiver impressions of pediatricians and staff who care for their children (Saxe-Custack et al., 2018). Caregivers have a distinct perspective from which to report experiences with their child's primary care, and these reports may be valid indicators of the quality of pediatric primary care (Seid et al., 2001). The purpose of the current study was to assess whether caregiver-reported perceptions of pediatric primary care changed after program exposure and whether perceptions of care varied by engagement in a pediatric FVPP.

## 2 Methods

### 2.1 Exposure: fruit and vegetable prescription program (FVPP)

As previously described, a large pediatric clinic in a low-income urban community introduced Michigan's first pediatric FVPP in 2016 (Saxe-Custack et al., 2018, Saxe-Custack et al., 2019b). The success of the program resulted in expansion to several pediatric and family practice clinics throughout Flint, Michigan and surrounding Genesee County (Saxe-Custack et al., 2021, Saxe-Custack et al., 2022). The program included pediatrician-issued $15 prescriptions for fresh produce that were distributed at every well-child or illness-related office visit a child attended. Prescriptions for fresh fruits and vegetables were built into the electronic medical record (EMR) system which allowed for easy distribution and reminded physicians to discuss fruit and vegetable consumption with patients. Pediatricians ordered prescriptions through the EMR system, printed on prescription paper, and distributed to all pediatric patients at office visits. Prescriptions were valid for 90 days and treated as vouchers redeemable only for fresh fruits and vegetables at a local farmers' market or mobile market/food hub that offers free home delivered produce boxes. Vendors returned redeemed prescriptions to the research team for payment and tracking.

### 2.2 Study design and population

This non-controlled longitudinal intervention trial included data from a consecutive sample of caregiver-child dyads at one large pediatric clinic in Flint as the pediatric FVPP was introduced (Saxe-Custack et al., 2022). For study enrollment, inclusion criterion was child age between 8 and 16 years (Saxe-Custack et al., 2022). Dyads were excluded if caregiver or child was not English speaking; legal guardian was absent at enrollment; child was previously exposed to the FVPP; child assent was refused; or sibling was previously enrolled.

Following consent and assent, caregiver–child dyads separately completed surveys to assess demographic characteristics and behavior at baseline, 12 months, and 24 months. Participants were compensated with gift cards in the amounts of $15, $40, and $50, respectively. Caregivers also reported perceptions of pediatric health care at the three time points. Specifically, caregivers completed the 23-item Parent's Perception of Primary Care which assessed caregiver perceptions of their child's pediatric health care (Seid et al., 2001). Trained research assistants provided detailed instructions regarding the study and survey completion. Caregivers and children completed follow-up surveys in-person at the pediatric office or remotely in response to phone calls, texts, and emails. All data were collected from February 2021 through May 2024 using a secure digital platform (Research Electronic Data Capture or REDCap). This study was approved by Michigan State University Institutional Review Board and was registered through clinicaltrials.gov [ID: NCT04767282] on February 23, 2021.

### 2.3 Measures

#### 2.3.1 Caregiver perceptions of primary care

Caregivers completed the 23-item Parent's Perceptions of Primary Care measure (P3C) which yielded a total score and subscale scores for continuity, access, contextual knowledge, communication, comprehensiveness, and coordination. Longitudinal continuity included two questions related to continuity of place of care (sample question is, “If there is one particular place you take your child for almost all his/her health care, how long has this been your child's place for healthcare?”). Access included four questions (sample question is, “If your child is sick, can you see the doctor within one day?”). Contextual knowledge included four questions (sample question is, “Do you feel your doctor knows your child's medical history?”). Communication included four questions (sample question is, “Does your doctor listen to you?”). Comprehensiveness included five questions (sample questions is, “Does your doctor talk to you about keeping your child heathy?”). Coordination of care included four questions (sample question is, “Do you feel your doctor communicates with other health providers about your child, when necessary?”). Except for the longitudinal questions, the response scale for the instrument was “never (0), sometimes (1), often (2), almost always (3), and always (4)”. These scores were transformed to a 0 to 100 scale, with 100 being the best/most desirable. The P3C serves as a practical, reliable, and valid measure of caregiver reports of pediatric primary care quality (Seid et al., 2001, Seid et al., 2003).

As distributions of the six subscales were strongly right skewed, scores were dichotomized to reflect either the caregiver reporting a score of 100 for the subscale or the caregiver reporting a score of less than 100 for the subscale. The total score of caregiver perceptions of primary care also had a maximum score of 100 and was less skewed than the subscales. Consequently, the continuous specification of the total score variable was retained.

#### 2.3.2 Demographics

Caregivers self-reported age, education, gender, household income, and participation in the Supplemental Nutrition Assistance Program (SNAP), and youth self-reported age, sex, and race. Year since baseline study enrollment was also recorded. Household income and SNAP use were modestly correlated. However, responses to survey questions related to household income were frequently missing (only 35% of participants responded), so household income was removed from the models to improve sample size.

#### 2.3.3 Prescription redemption

To explore whether trends in perception of pediatric primary care varied according to FVPP engagement, analyses were stratified by redemption status. Program engagement was defined as redemption of least one $15 prescription during the 24-month study period, and no engagement was defined as no prescription redemption during the 24-month study period.

### 2.4 Statistical analysis

Means (standard deviation) and frequencies (percentages) were employed to describe the baseline characteristics and outcomes at baseline, 12 months, and 24 months. Outcome means and percentages for the entire sample were determined, then stratified by FVPP engagement.

In order to evaluate absolute magnitude of the overall and engagement group-level effects over time, we estimated marginal means (continuous) and predicted probabilities (binary) of the outcomes at each time point using linear mixed models and generalized linear mixed models (logit link and binomial distribution) with participant-specific random intercepts. Time was characterized as a three-level categorical variable (baseline, 12 months, and 24 months). Models were adjusted for adult age, child age, child sex, child race, adult educational attainment, adult gender, participation in SNAP, and year of baseline encounter. Unstratified models of the entire sample's trends included redemption status as a covariate. To estimate marginal means and predicted probabilities stratified by FVPP engagement, linear mixed models and generalized linear mixed models were generated for participants who redeemed at least one prescription (engaged) and for those who redeemed no prescriptions (not engaged).

To estimate a *p*-value for the interaction between time and FVPP engagement, researchers generated linear mixed models and generalized linear mixed models with an interaction term for time x redemption status. A p-value below 0.05 was considered statistically significant. All analyses were conducted using SAS 9.4 (SAS Institute, Cary NC, USA).

#### 2.4.1 Sensitivity analyses

To evaluate the impact of redemption timing and missingness, we conducted two sensitivity analyses. The first used a variable for redemption status that included four categories: did not redeem, redeemed at least one prescription between baseline and 12 months only, redeemed at least one prescription between 12 months and 24 months only, and redeemed at least one prescription between baseline and 12 months and at least one prescription between 12 and 24 months. This variable was included as a covariate in multivariable models for the overall effect of time on caregiver satisfaction as sample sizes were too small for some categories to stratify models. To ensure the impact of missingness did not change interpretation of the results, the second analysis limited the sample to individuals with complete P3C scores at baseline and 24 months, missingness was allowed at 12 months to preserve sample size.

## 3 Results

A total of 253 dyads enrolled in the study. Of those, 247 (97.6%) had complete data for all covariates for at least one time point and were included in the analyses. Among youth participants, 134 (54.3%) were aged 8 to 11 years, and 125 (50.6%) identified as female. Mean age of caregivers was 38.2 ± 9.4 years, and 222 (89.9%) were female. A total of 100 (40.5%) caregivers reported having a high school diploma or less. As shown in Table 1, 162 caregivers (34%) reported participation in SNAP in the previous 12 months, and 103 (41.7%) redeemed at least one prescription during the 24-month study period (range of redemption was 1–15 prescriptions). Total number of prescriptions received by study participants ranged from 1 to 18 (median number received was 3). Differences in the sample when stratified by FVPP engagement included child age, child race, adult age, participation in SNAP, and number of prescriptions received.

**Table 1:** Descriptive Characteristics of Caregiver-Child Dyads Overall and Stratified by Fruit and Vegetable Prescription Program Engagement at One Pediatric Clinic in Flint Michigan USA, 2021–2022 (*n* = 247).

Descriptive statistics	Overall n (%)	Engaged n (%)	Not Engaged n (%)	Χ^2^*p*-value
**Child Age**				0.20
Child Age 8–9 Years Old	62 (25.1)	22 (21.4)	40 (27.8)	
Child Age 10–11 Years Old	72 (29.2)	27 (26.2)	45 (31.3)	
Child Age > 11 Years Old	113 (45.8)	54 (52.4)	59 (41.0)	

**Child Sex**				0.77
Female	125 (50.6)	51 (49.5)	74 (51.4)	
Male	122 (49.4)	52 (50.5)	70 (48.6)	

**Child's Race**				0.17
Black (alone or in combination with other race)	186 (75.3)	73 (70.9)	113 (78.5)	
Not Black	61 (24.7)	30 (29.1)	31 (21.5)	

**Adult Age Mean ± SD**	38.2 ± 9.4	40.7 ± 10.1	36.4 ± 8.5	<0.01⁎

**Adult Education**				0.66
HS Diploma or less	100 (40.5)	44 (42.7)	56 (38.9)	
Some College or Certification	97 (39.3)	37 (35.9)	60 (41.7)	
Associate's Degree or Higher	50 (20.2)	22 (21.4)	28 (19.4)	

**Adult Gender**				0.86
Female	222 (89.9)	93 (90.3)	129 (89.6)	
Male	25 (10.1)	10 (9.7)	15 (10.4)	

**Participation in SNAP**				0.22
Yes	162 (65.6)	63 (61.2)	99 (68.8)	
No	85 (34.4)	40 (38.8)	45 (31.2)	

**Year**				0.27
2021	158 (64.0)	70 (68.0)	88 (61.1)	
2022	89 (36.0)	33 (32.0)	56 (38.9)	

**Prescriptions Received**				
Median (q25, q75)	3 (2, 5)	4 (3, 7)	3 (2,4)	<0.01

**Engaged in Fruit and Vegetable Prescription Program**				
Yes	103 (41.7)			
No	144 (58.3)			

⁎ indicates p-value from analysis of variance (ANOVA) f-test `indicates p-value from Wilcoxon Two-Sample Test.

As shown in Table 2, the unadjusted analyses demonstrated that most outcomes remained stable over time for the entire sample. Among those who redeemed at least one fruit and vegetable prescriptions (engaged), the frequency that caregivers rated pediatric primary care at 100 improved for the following subscales: continuity, contextual knowledge, communication, comprehensiveness, and coordination of care. Among those who did not redeem at least one prescription (not engaged), unadjusted frequencies of reporting a score of 100 on these subscales was either stable or declined over the 24-month period. The unadjusted mean total score of P3C increased over time for participants engaged in the FVPP by nearly 5 points, whereas the unadjusted mean score for non-engaged participants decreased by 6.4 points.

**Table 2:** Unadjusted Frequencies and Means for Caregiver Perceptions of Primary Pediatric Care Outcomes at Baseline, 12-Month Follow-Up, and 24-Month Follow-Up, Stratified by Fruit and Vegetable Prescription Program Engagement for One Pediatric Clinic in Flint Michigan USA, 2021–2024 (n = 247).

	Baseline Score of 100 n (percent/probability)	12-Month Score of 100 n (percent/probability)	24-Month Score of 100 n (percent/probability)
Longitudinal Continuity	98 (39.8)	52 (38.0)	48 (42.5)
Longitudinal Continuity No Engagement	60 (41.7)	22 (34.4)	21 (37.5)
Longitudinal Continuity Engagement	38 (37.3)	30 (41.1)	27 (47.4)
Missing	1	110	134

Access	82 (33.2)	28 (20.6)	28 (24.8)
Access No Engagement	54 (37.5)	14 (22.2)	19 (33.9)
Access Engagement	28 (27.2)	14 (19.2)	9 (15.8)
Missing	0	111	134

Contextual Knowledge	130 (52.6)	71 (51.8)	57 (50.4)
Contextual Knowledge No Engagement	79 (54.9)	29 (45.3)	25 (44.6)
Contextual Knowledge Engagement	51 (49.5)	42 (57.5)	32 (56.1)
Missing	0	110	134

Communication	159 (64.4)	82 (59.9)	71 (62.8)
Communication No Engagement	98 (68.1)	34 (53.1)	31 (55.4)
Communication Engagement	61 (59.2)	48 (65.6)	40 (70.2)
Missing	0	110	134

Comprehensiveness	117 (47.4)	59 (43.1)	56 (49.6)
Comprehensiveness No Engagement	75 (52.1)	27 (42.2)	24 (42.9)
Comprehensiveness Engagement	42 (40.8)	32 (43.8)	32 (56.2)
Missing	0	110	134

Coordination	135 (57.7)	73 (54.9)	69 (63.3)
Coordination No Engagement	84 (61.3)	30 (48.4)	28 (51.9)
Coordination Engagement	51 (52.6)	3 (60.6)	41 (74.6)
Missing	13	114	138

Unadjusted Mean Total Score (std)	81.4 (17.8)	81.1 (17.0)	80.5 (20.8)
Unadjusted Mean Total Score (95% CI) No Engagement	82.5 (18.0)	79.8 (17.4)	76.1 (23.8)
Unadjusted Mean Total Score (95% CI) Engagement	79.8 (17.4)	82.2 (16.6)	84.7 (16.4)
Missing	0	110	134

As shown in Table 3, trends in predicted probabilities of caregivers reporting the maximum score of 100 for P3C subscales were stable and largely not significant, but consistent trends emerged based on redemption status. Trends in predicted probabilities of P3C scores of 100 for continuity, contextual knowledge, communication, comprehensiveness, and coordination of care increased among participants engaged in the FVPP and generally stayed stable or decreased for participants not engaged in the FVPP. Calculated *p*-values for the interaction between time and redemption status were significant for the coordination of care subscale (*p* = 0.02) and total P3C score (*p* = 0.01), and marginally significant for the communication subscale (*p* = 0.06).

**Table 3:** Predicted Probabilities and Mean Scores for Caregiver Perceptions of Primary Care Outcomes at Baseline, 12-Month Follow-Up, and 24-Month Follow-Up, Stratified by Fruit and Vegetable Prescription Program Engagement for One Pediatric Clinic in Flint Michigan USA, 2021–2024 (n = 247).

	Marginal Probability of Giving 100% at Baseline	Marginal Probability of Giving 100% at 12 months	Marginal Probability of Giving 100% at 24 months	Interaction p-value
Longitudinal Continuity	0.32	0.30 *p* = 0.71	0.35 *p* = 0.54	
Longitudinal Continuity No Engagement	0.34	0.27 *p* = 0.35	0.32 *p* = 0.80	0.33
Longitudinal Continuity Engagement	0.31	0.35 *p* = 0.54	0.42 *p* = 0.21	

Access	0.39	**0.25** **p = 0.01**	0.30 *p* = 0.14	
Access No Engagement	0.42	**0.24** ***p* = 0.03**	0.37 p = 0.54	0.51
Access Engagement	0.34	0.23 *p* = 0.21	0.19 p = 0.14	

Contextual Knowledge	0.50	0.48 *p* = 0.69	0.47 *p* = 0.59	
Contextual Knowledge No Engagement	0.50	0.38 *p* = 0.17	0.38 *p* = 0.18	0.17
Contextual Knowledge Engagement	0.52	0.60 *p* = 0.32	0.59 *p* = 0.48	

Communication	0.65	0.59 *p* = 0.30	0.64 *p* = 0.78	
Communication No Engagement	0.70	**0.53** ***p* = 0.04**	0.57 *p* = 0.12	0.06
Communication Engagement	0.60	0.67 *p* = 0.47	0.73 *p* = 0.16	

Comprehensiveness	0.45	0.41 *p* = 0.41	0.48 p = 0.69	
Comprehensiveness No Engagement	0.50	0.40 p = 0.21	0.40 *p* = 0.27	0.10
Comprehensiveness Engagement	0.40	0.42 *p* = 0.76	0.58 p = 0.06	

Coordination	0.55	0.50 *p* = 0.44	0.61 *p* = 0.39	
Coordination No Engagement	0.59	0.44 *p* = 0.09	0.49 *p* = 0.26	**0.02**
Coordination Engagement	0.50	0.57 p = 0.44	**0.75** **p = 0.01**	
	Marginal Mean Total Score at Baseline	Marginal Mean Total Score at 12 Months	Marginal Mean Total Score at 24 months	
Adjusted Mean Total Score	81.1	80.1 p = 0.54	80.0 *p* = 0.50	
Adjusted Mean Total Score No Engagement	81.9	78.9 *p* = 0.22	**75.8** **p = 0.02**	**0.01**
Adjusted Mean Total Score Engagement	80.7	81.9 *p* = 0.53	84.8 *p* = 0.07	

Models allow for missingness of outcome variables. Models adjusted for age of adult, age of child, child's biological sex, child's race, adult educational attainment, adult gender, participation in SNAP, and year of baseline encounter. Models used were linear mixed models (total score) and generalized linear mixed models (logistic regression, subscale scores of 100 versus less than 100). **Bolding** indicates a time point statistically different from baseline estimate (*p* < 0.05). p-values in 12 month and 24 month estimate cells represent the contrast between the time point estimate and the baseline estimate. The interaction *p*-values were calculated from models that included an interaction term for time and program engagement.

The sensitivity analyses for redemption timing did not meaningfully change the effect estimates of P3C at each time point (Supplement Table S1). The sensitivity analyses for missingness altered effect estimates but did not ultimately alter patterns of P3C (Supplement Table S2).

## 4 Discussion

Although caregiver perceptions of child's primary care remained largely stable for the entire sample across 24 months, important differences emerged when stratifying by engagement in the FVPP. Those who redeemed at least one fruit and vegetable prescription reported significant improvements in overall perceptions of their child's primary care from baseline to 24 months, with notable gains across the coordination of care subscale. In contrast, those who did not redeem at least one fruit and vegetable prescription demonstrated significant declines in perceptions of primary care, particularly in relation to communication and access to care at the 12-month timepoint. These findings suggest that participation in the pediatric FVPP may meaningfully enhance families' experience within clinical settings. The act of redeeming a prescription may reflect or promote stronger engagement in both the program and healthcare system, potentially reinforcing trust, perceived support, and feelings of being cared for by the clinical team (Auvinen et al., 2022; Hager et al., 2023; Rhodes et al., 2024).

Findings underscore the potential of pediatric produce prescription programs to improve or strengthen caregiver-provider relationships. Families who engaged in the FVPP reported greater satisfaction with their child's primary care when compared to families who did not engage, particularly in relation to coordination of pediatric care and communication with providers. This suggests that the FVPP may serve as an important connector between families and their primary care team. Results support earlier qualitative research that suggested caregivers perceive this pediatric FVPP as both a nutritional support initiative and a confirmation of physician investment in their child's broader well-being (Saxe-Custack et al., 2018). Observed patterns indicate that integrating resource-based interventions into pediatric primary care may strengthen both family engagement and perceived quality of health care which are critical to improving health equity and partnerships between providers and families (Council on Children with Disabilities and Medical Home Implementation Project Advisory Committee, 2014).

Declining caregiver perceptions of primary care were observed among families who did not redeem prescriptions. This highlights a subgroup that may face substantial barriers to accessing health-promoting programs (e.g., limited transportation, food access challenges, or medical mistrust). Challenges to engagement in the current FVPP have been previously identified by caregivers (Saxe-Custack et al., 2024). Efforts are underway to address these obstacles to participation, including expanding the FVPP to include grocery stores as redemption sites and developing a comprehensive FVPP education plan at pediatric clinics. Future research will investigate the impact of these efforts on program engagement.

Previous research has identified disparities in pediatric health care across socioeconomic and racial groups (Chokshi, 2018; Seid et al., 2003; Weech-Maldonado et al., 2001) that often lead to preventable differences in health outcomes, with marginalized children facing disproportionate burdens (Shahidullah et al., 2023). A growing body of research indicates an increase in medical mistrust among caregivers across the US (Tekeste et al., 2025). Importantly, caregivers who are African American, or who reside in marginalized communities, frequently report higher levels of mistrust toward pediatric providers and healthcare institutions when compared to caregivers who are White (Armstrong et al., 2007). Studies have demonstrated that higher levels of mistrust are associated with lower satisfaction of care, reduced adherence to medical recommendations, and delays in seeking care (Jacobs et al., 2006; Tekeste et al., 2025). This also collectively underscores the vital role trust plays in shaping healthcare engagement and outcomes for marginalized families (Jacobs et al., 2006). Previous qualitative work on the current FVPP has suggested that caregivers are grateful to physicians for providing a tangible resource to help families achieve a healthy diet, rather than simply telling them to do so (Saxe-Custack et al., 2018). This FVPP is widespread in Flint, Michigan and offered at clinics that serve primarily low-income families. Although previous research has documented the success of this FVPP in improving food security, dietary patterns, and health markers in youth (Saxe-Custack et al., 2019a, Saxe-Custack et al., 2021, Saxe-Custack et al., 2022, Saxe-Custack et al., 2025), the current study further suggests that the FVPP may be an effective resource to improve perceptions of pediatric primary care and address mistrust among families.

Although efforts have been made to reduce disparities in pediatric health care, little is known about the impacts of nutrition programs that are designed, in whole or in part, to combat such disparities. Beyond standard nutrition support, FVPPs address enduring environmental barriers related to access and cost of fresh produce. Furthermore, consistent messages from pediatricians reinforce the significant role of fresh, nutritious foods in supporting health and preventing disease. Early research has suggested that caregivers whose children were exposed to this year-round FVPP not only perceived the program as effective in improving child dietary patterns and household food security but also appreciate that pediatricians provide foods to support nutrition and health (Saxe-Custack et al., 2018). The current study highlights novel effects of pediatric FVPPs that have not been previously reported.

This study has several limitations. The study was small and limited to one geographic area, so results may not be generalizable to a broader population. Although study participants were demographically similar, it is possible that confounding factors were not captured between the FVPP engagement groups that contributed to differences in caregiver perceptions of primary care, particularly household or family income. Participation in SNAP, which has income requirements, was used as a proxy for family income due to family income missingness. This may have failed to fully capture economic hardship for non-SNAP participating families.

## 5 Conclusions

The current study is the first to fill gaps in evidence about the potential effects of FVPPs in relation to caregiver perceptions of their child's primary care. Unique in its focus, this study extends evaluation of the current pediatric FVPP beyond feasibility and preliminary effectiveness and suggests the potential of the program to positively influence caregiver perceptions of pediatric healthcare.

## CRediT authorship contribution statement

**Amy Saxe-Custack:** Writing – original draft, Supervision, Project administration, Methodology, Funding acquisition, Conceptualization. **Diana Haggerty:** Writing – original draft, Visualization, Validation, Formal analysis. **Gayle Shipp:** Writing – review & editing. **Jenny LaChance:** Writing – review & editing.

## Funding

This work was supported by the Eunice Kennedy Shriver National Institute of Child Health and Human Development of the National Institutes of Health [grant number R01HD102527]. The funders had no role in the design of the study; in the collection, analysis, or interpretation of data; in the writing of the manuscript; or in the decision to publish the results.

## Declaration of competing interest

The authors declare that they have no known competing financial interests or personal relationships that could have appeared to influence the work reported in this paper.

## Data Availability

The final de-identified dataset will be shared with a National Institutes of Health-supported data repository. This dataset will be removed of all identifiers. Information will be shared under a strict data-sharing agreement that provides for: (1) a commitment to using data only for research purposes and not to identify any individual participant; (2) a commitment to securing the data using appropriate computer technology; and (3) a commitment to destroying or returning the data after analyses are completed.

## Appendix A Supplementary data

Supplementary material The supplement tables contain sensitivity analyses that evaluate the impact of fruit and vegetable prescription program redemption timing and missingness. Supplementary material
