# Supplementary material for: Influence of a pediatric fruit and vegetable prescription program in Flint Michigan on caregiver perceptions of pediatric health care
Source: Prev Med Rep. 2026 Feb 20;64:103421. doi: 10.1016/j.pmedr.2026.103421 (PMC12954189; doi:10.1016/j.pmedr.2026.103421)
Supplement: Supplementary material — The supplement tables contain sensitivity analyses that evaluate the impact of fruit and vegetable prescription program redemption timing and missingness. [file mmc1.docx]

**Supplement Table S1. Predicted Probabilities and Mean Scores for Caregiver Perceptions of Primary Care Outcomes at Baseline, 12-Month Follow-Up, and 24-Month Follow-Up, Adjusted for Redemption Using Four Categories at One Pediatric Clinic in Flint Michigan USA, 2021-2024 (n = 247)**

|  | **Marginal Probability of Giving 100% at Baseline (95% CI)** | **Marginal Probability of Giving 100% at 12 months (95% CI)** | **Marginal Probability of Giving 100% at 24 months (95% CI)** |
| --- | --- | --- | --- |
| **Longitudinal Continuity** | 0.31 (0.21, 0.44) | 0.29 (0.19, 0.43) | 0.35 (0.22, 00.50) |
| **Access** | 0.37 (0.25, 0.50) | 0.23 (0.13, 0.36) | 0.28 (0.13, 0.43) |
| **Contextual Knowledge** | 0.50 (0.37, 0.63) | 0.47 (0.33, 0.62) | 0.46 (0.31, 0.61) |
| **Communication** | 0.65 (0.52, 0.77) | 0.59 (0.43, 0.73) | 0.63 (0.47, 0.77) |
| **Comprehensiveness** | 0.44 (0.31, 0.57) | 0.39 (0.26, 0.54) | 0.46 (0.31, 0.61) |
| **Coordination** | 0.55 (0.41, 0.68) | 0.50 (0.35, 0.65) | 0.60 (0.44, 0.74) |
| **Adjusted Mean Total Score** | 80.41 (76.16, 84.66) | 79.40 (74.73, 84.07) | 79.18 (74.36, 84.00) |

**Supplement Table S2. Predicted Probabilities and Mean Scores for Caregiver with Complete Baseline and 24-month Perceptions of Primary Care Outcomes at Baseline, 12-Month Follow-Up, and 24-Month Follow-Up, Stratified by Fruit and Vegetable Prescription Program Engagement for One Pediatric Clinic in Flint Michigan USA, 2021-2024 (n=113)**

|  | **Marginal Probability of Giving 100% at Baseline** | **Marginal Probability of Giving 100% at 12 months** | **Marginal Probability of Giving 100% at 24 months** | **Interaction**  **p-value** |
| --- | --- | --- | --- | --- |
| **Longitudinal Continuity** | 0.24 | 0.31  p = 0.29 | 0.31  p = 0.23 |  |
| **Longitudinal Continuity**  **No Engagement** | 0.28 | 0.19  p = 0.33 | 0.26  p = 0.82 | 0.06 |
| **Longitudinal Continuity Engagement** | 0.20 | 0.43  p = 0.02 | 0.37  p = 0.05 |  |
|  |  |  |  |  |
| **Access** | 0.41 | 0.23  p = 0.02 | 0.28  p = 0.08 |  |
| **Access**  **No Engagement** | 0.53 | 0.31  p = 0.06 | 0.40  p = 0.22 | 0.87 |
| **Access**  **Engagement** | 0.20 | 0.09  p = 0.15 | 0.09  p = 0.14 |  |
|  |  |  |  |  |
| **Contextual Knowledge** | 0.50 | 0.50  p = 0.99 | 0.47  p = 0.66 |  |
| **Contextual Knowledge**  **No Engagement** | 0.49 | 0.45  p = 0.74 | 0.39  p = 0.37 | 0.75 |
| **Contextual Knowledge**  **Engagement** | 0.55 | 0.58  p = 0.80 | 0.57  p = 0.83 |  |
|  |  |  |  |  |
| **Communication** | 0.64 | 0.63  p = 0.91 | 0.66  p = 0.76 |  |
| **Communication**  **No Engagement** | 0.64 | 0.63  p = 0.93 | 0.60  p = 0.68 | 0.57 |
| **Communication Engagement** | 0.68 | 0.66  p = 0.88 | 0.77  p = 0.33 |  |
|  |  |  |  |  |
| **Comprehensiveness** | 0.42 | 0.43  p = 0.92 | 0.49  p = 0.39 |  |
| **Comprehensiveness**  **No Engagement** | 0.49 | 0.52  p = 0.79 | 0.44  p = 0.68 | 0.23 |
| **Comprehensiveness Engagement** | 0.43 | 0.42  p = 0.93 | 0.62  p = 0.09 |  |
|  |  |  |  |  |
| **Coordination** | 0.56 | 0.55  p = 0.91 | 0.63  p = 0.29 |  |
| **Coordination**  **No Engagement** | 0.60 | 0.51  p = 0.44 | 0.51  p = 0.38 | 0.07 |
| **Coordination**  **Engagement** | 0.50 | 0.58  p = 0.48 | 0.77  p = 0.01 |  |
|  |  |  |  |  |
| **Adjusted mean total score** | 81.6 | 81.6  p = 0.97 | 80.6  p = 0.57 |  |
| **Adjusted Mean Total Score No Engagement** | 79.30 | 77.42  p = 0.58 | 73.46  p = 0.06 | 0.04 |
| **Adjusted Mean Total Score Engagement** | 84.03 | 85.73  p = 0.48 | 87.65  p = 0.12 |  |
